# Getting the flu: 5 key facts about influenza virus evolution

**DOI:** 10.1371/journal.ppat.1006450

**Published:** 2017-08-24

**Authors:** Katherine E. E. Johnson, Timothy Song, Benjamin Greenbaum, Elodie Ghedin

**Affiliations:** 1 Center for Genomics & Systems Biology, Department of Biology, New York University, New York, New York, United States of America; 2 Tisch Cancer Institute, Departments of Genetics and Genomics, Medicine, Oncological Sciences, and Pathology, Icahn School of Medicine of Mt Sinai, New York, New York, United States of America; 3 Department of Epidemiology, College of Global Public Health, New York University, New York, New York, United States of America; University of Kentucky, UNITED STATES

## Introduction

The year 1918 saw the most famous influenza pandemic—a worldwide epidemic that caused nearly 50 million deaths—when an H1N1 influenza A virus of partial avian origin infected over one-third of the world’s population. Although its exact origins are still under debate, World War I and trade routes are thought to have aided in the circulation of the virus worldwide [1]. Within the last century, there have been 4 pandemics caused by influenza A, with the most recent in 2009 when a swine-like H1N1 subtype virus entered the human population. Increased whole genome sequencing and computational methods have accompanied improved surveillance of bird populations [2] and of human households and communities [3]. This allows for analysis of large datasets and the ability to glimpse into influenza evolution. A better understanding of the dynamics of influenza A and B evolution will bring insight into flu transmission, adaptation to new hosts, and outbreak potential.

## How does flu evolve? By making mistakes and reshuffling genes

The 2 main influenza types that infect humans, A and B, are part of the Orthomyxoviridae family, which is characterized by segmented negative-sense RNA genomes. These viruses replicate using an RNA-dependent RNA polymerase that lacks proofreading capability. This means that errors that occur during replication produce a diversity of influenza mutants—also called variants—leading to populations of viruses that are often referred to as quasispecies [4]. These mutations allow viruses to adapt to changing environments, leading to continuous selective turnover of influenza variants.

**Fig 1:**
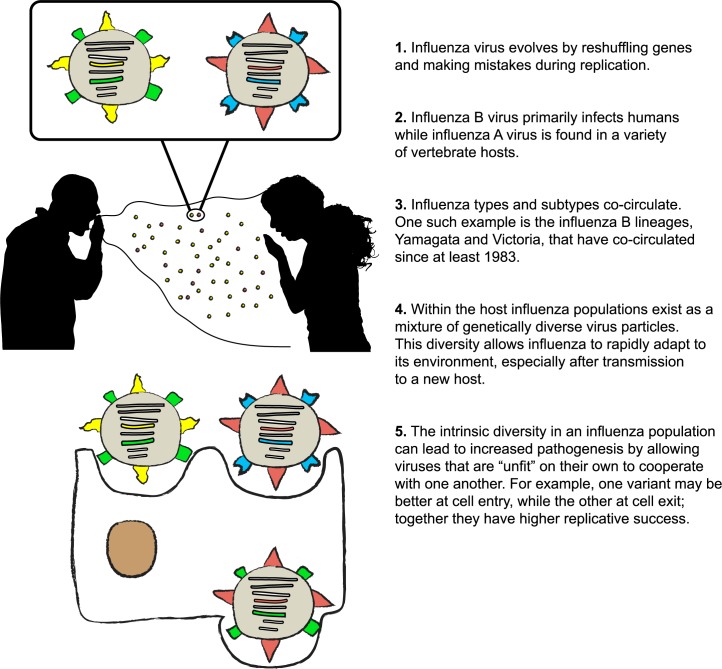
Five key facts about influenza virus evolution.

The segmented structure of the influenza genome facilitates gene exchange between different influenza strains that have infected the same cell (Fig 1). Gene reassortment has been observed in both influenza A and B. Because of the large range of hosts that can get infected with influenza A, reassortment among influenza A viruses can lead to the introduction of subtypes that are antigenically novel for the human population. Intersubtype reassortment has the potential to cause a pandemic; this occurred in the pandemics of both 1957 and 1968 when a reassortment event took place between avian and human influenza viruses [5]. However, because of genetic dissimilarities and incompatibility between interacting proteins, intersubtype exchange may produce gene rearrangements that do not efficiently transmit among humans, therefore reducing the fitness of the virus [6–8]. Similarly, reassortment between lineages of the same subtype, or intrasubtype reassortment, is limited by genetic compatibilities between the segments of the viruses [9, 10]. Studies measuring intrasubtype gene exchange found that reassortment between lineages of the same subtype is common and can lead to severe epidemics [11]. While both mutations and gene reassortments can produce antigenic variants, gene exchange produces a rapid change in a virus’s antigenicity (antigenic shift), whereas mutations cause a more gradual change (antigenic drift). However, the combination of mutations with gene reassortments can assist in segment exchange by allowing the gene to adapt to its new genetic environment [12].

## Who gets the flu? Influenza has a wide host range

Humans are not the only animals to get influenza infections. Influenza A has a variety of vertebrate hosts, including birds, horses, pigs, and bats [13]. In contrast, humans are almost the exclusive host for influenza B viruses—with some surprising infections in seals that have been documented [14]. Subtypes of influenza A virus are named after the 2 glycoproteins found on the surface of the virus, hemagglutinin (HA) and neuraminidase (NA), which are respectively essential for entry into and release from the host cell. Aquatic birds are thought to be the primary reservoir for influenza A because they can be infected with nearly every subtype of the virus, except for the recently discovered subtypes H17N10 and H18N11 found in bats [15]. With the rapid evolution of influenza, the increase of influenza host range remains a cause for concern, especially in mammals, because of the higher probability of a novel strain arising that can have devastating effects on the human population.

It is uncommon for humans to be directly infected with avian flu viruses; it typically requires the infection of an intermediate host in which the virus undergoes adaptation leading to variants that can subsequently more easily transmit to humans. These hosts are often referred to as “mixing vessels,” as seen for pigs, which can be infected with avian and human influenza viruses, producing a favorable environment for gene exchange between strains [16]. With the proper gene rearrangements, new viruses can arise and cross species boundaries, infect humans, and cause a pandemic, as witnessed in 1957, 1968, and 2009.

## What gets around? Types, subtypes, and strains of flu cocirculate

Recently, the interplay between influenza transmission and evolution has been of particular interest in characterizing interspecies transmission, expansion of host range, and the diversity of viruses circulating. Cocirculation of different influenza virus strains contributes to the continued evolution of flu by increasing the possibility of segment exchange occurring between lineages within subtypes. Although influenza A subtypes H1N1 and H3N2 have been cocirculating since 1977—with the 2009 pandemic H1N1 strain [17], replacing the circulating seasonal H1N1—intersubtype reassortment between H1N1 and H3N2 is rare [18]. Influenza B lineages, Yamagata and Victoria, have cocirculated since at least 1983 [19] and have been reassorting extensively [20].

Influenza A appears to be evolving under pressure to avoid CpG-containing oligonucleotides, possibly due to innate immune recognition, in a manner less present in influenza B evolution [21, 22]. Moreover, influenza B lineages have been shown to have lower rates of antigenic drift when compared to influenza A subtypes [23]. However, distinct patterns in the rate of antigenic drift between the subtypes of influenza A and lineages of influenza B have emerged. Influenza A H3N2 and influenza B Victoria lineage undergo a more rapid antigenic change; thus, old strains die out while new strains appear, lowering diversity [18, 23, 24]. In contrast, influenza A H1N1 and influenza B Yamagata lineage undergo a slower rate of antigenic drift, which allows for more lineages to cocirculate at one time with reduced competition [18, 23, 24]. A recent model comparing antigenic drift with transmission efficiency found that higher rates of transmission result in accelerated antigenic drift [25].

## What’s worse than 1 virus? A swarm of viruses

Because of the error-prone RNA polymerase, influenza populations exist as a mixture of genetically diverse viral particles. Interactions among the variants could contribute to the overall success and fitness of the population. The high diversity seen within an infection leads to influenza adapting rapidly to a new environment. The analysis of deep sequencing data from infected individuals in chains of transmission has allowed the quantification of transmission events. It was observed that a genetic mix of viruses is transmitted to susceptible hosts, with minor variants tagging along with the dominant strain. It is thus feasible that while the influenza vaccine targets the dominant strain, minor variants continue to transmit, undergoing positive selection and becoming the dominant strain in subsequent transmissions [26].

When looking further at intrahost evolution of the flu, studies find that moderately deleterious mutations in influenza may remain in the population over several chains of transmission [27, 28]. Although these mutations may not be systematically removed through purifying, or negative, selection, they also do not become fixed within the population [29]. However, computational models show that deleterious mutations can impact the overall evolutionary trajectory of influenza by restricting the rate of antigenic change of the virus [30, 31].

## How much worse than 1 virus are 2 viruses? They can be more than twice as bad

A recent study on H3N2 found that cooperation could clearly occur between 2 different H3N2 variants. With 1 variant excelling in cell entry and the other excelling in cell exit, the viral population had a higher replicative success when the 2 variants were mixed than when they were separate [32]. Similar results were found when looking at the expression of influenza proteins. Although individual virions that lack a fundamental protein may appear noninfectious, when they are part of a viral population, they can contribute by complementation-dependent protein expression to increase the fitness of the population as a whole [33]. Influenza virus clones can also compete with one another, a phenomenon that has been used to explain the appearance of selective sweeps in influenza’s antigenic evolution [34]. While cooperation is associated with the division of labor between variants (in the above example, between cellular entry and exit), competition occurs when multiple members of a population independently develop beneficial mutations. This scenario, amongst other things, can create an arms race between members of the population. Thus, the intrinsic diversity and loose transmission bottlenecks in circulating influenza populations can facilitate an increase in pathogenesis by allowing viral variants to cooperate with one another or by increasing the number of beneficial mutations appearing in cocirculating clones.

Clearly, influenza evolution is a multilayered process, and much work needs to be done to fully capture its richness. Such work requires mathematical tools and next-generation sequencing technologies to operate in concert to capture the full breadth of influenza transmission. Effective vaccination strategies would require a better understanding of the complex interplay of multivariant transmission dynamics, inter- and intrahost viral evolution, and the interaction of diverse strains with the host immune system. Only then will a full picture of influenza’s evolutionary dynamics emerge.
